# SUPG-Stabilized Virtual Element Method for Optimal Control Problem Governed by a Convection Dominated Diffusion Equation

**DOI:** 10.3390/e23060723

**Published:** 2021-06-05

**Authors:** Qiming Wang, Zhaojie Zhou

**Affiliations:** School of Mathematics and Statistics, Shandong Normal University, Jinan 250000, China; wangqiming_sdnu@126.com

**Keywords:** optimal control, convection dominated diffusion equation, SUPG stabilized VEM, a priori error estimate

## Abstract

In this paper, the streamline upwind/Petrov Galerkin (SUPG) stabilized virtual element method (VEM) for optimal control problem governed by a convection dominated diffusion equation is investigated. The virtual element discrete scheme is constructed based on the first-optimize-then-discretize strategy and SUPG stabilized virtual element approximation of the state equation and adjoint state equation. An a priori error estimate is derived for both the state, adjoint state, and the control. Numerical experiments are carried out to illustrate the theoretical findings.

## 1. Introduction

In this paper, we aim to discuss a priori error analysis of SUPG stabilized virtual element method (VEM) for the optimal control problem governed by the convection dominated diffusion equation. We consider the following optimal control problem:

(1) $$\begin{array}{c} \min_{u\in U_{ad}}J\left(y,u\right):=\frac{1}{2}∥y-y_{d}{∥}^{2}+\frac{\gamma }{2}{∥u∥}^{2} \end{array}$$

subject to

(2) $$\begin{array}{c} \left\{\begin{array}{ccc} -\nabla \cdot (\varepsilon \nabla y)+\beta (x)\cdot \nabla y+\delta y & =f+u\; & \mathrm{in}\;\Omega ,\\ y & =0\; & \mathrm{on}\;\Gamma , \end{array}\right. \end{array}$$

where J(y,u) is the objective functional, $y_{d}\in L^{2}\left(\Omega \right)$ is the desired state, and γ>0 is the regularization parameter. 0<ε≪1 represents constant diffusion coefficient and $\beta \in {\left[W^{1,\infty }\left(\Omega \right)\right]}^{2}$ with ∇·β=0 is the transport advective field. δ>0 is a constant. $f\in L^{2}\left(\Omega \right)$ is the volume source term. $\Omega \subset R^{2}$ is a bounded domain with Γ=∂Ω. The control constraint set is given by

$$U_{ad}=\left\{u\in L^{2}\left(\Omega \right):\;u_{a}\leq u\left(x\right)\leq u_{b}\;\;\mathrm{a.e.}\mathrm{in}\;\Omega \;\;\mathrm{with}\;\;u_{a},u_{b}\in R\;\;\mathrm{and}\;\;u_{a}\leq u_{b}\right\}.$$

Optimal control problems governed by the convection dominated diffusion equation have many applications in real life, such as the air pollution problem ([1]) and waste water treatment problem ([2]). It is well known that a characteristic of convection-dominated equation is that the solutions may have the sharp boundary and interior layers when the coefficient of the convection field is relatively large. Since numerical methods without any treatment do not work well in this case, various robust schemes such as SUPG formulation, residual-free bubbles methods, and discontinuous Galerkin methods for convective dominance equations have been developed. For the numerical approximation of the convection diffusion optimal control problem, we refer to [3,4,5,6,7,8,9,10].

VEM can be regarded as a generalization of the finite element method (FEM) to general polygonal and polyhedral meshes, and it is originally introduced in [11] as a $C^{0}$-conforming method for solving the two-dimensional Poisson equation. Thus far, VEM has been used in a variety of problems, such as elliptic problem ([12,13,14]), parabolic problem ([15]), and Stokes problem ([16,17]). The method performs well in geometrically complex domains [18] and with badly shaped polygonal elements [19]. In fact, the underlying virtual element space can be seen as the finite element space plus some suitable non-polynomial functions, which are the solutions of PDE problems inside each element. Compared with SUPG-FEM, SUPG-VEM provides great flexibility for us to use arbitrary polygonal meshes (even non-convex). SUPG-VEM also has great advantages for adaptive refinement. For instance, locally adapted meshes do not require any local post processing because polygonal meshes are allowed, and any limitations caused by maximum angle conditions or mesh distortion are eliminated. In addition, we do not need to add additional degrees of freedom for hanging nodes during adaptive refinement, since we can just treat the hanging nodes as new nodes. However, there are not many studies on SUPG-VEM of convection dominant problems. Cangiani et al. first studied the non-consistent SUPG-VEM problem of convection-dominated diffusion in [20]. Subsequently, SUPG-stabilized conforming and non-conforming VEMs are presented in [21,22]. However, the stability and convergence analysis in [21,22] is not uniform in the diffusion/convection parameters, and a small enough mesh size is needed for analysis. Recently, Beirão da Veiga et al. discussed a robustness analysis of SUPG-stabilized virtual elements for diffusion–convection problems in [23]. By slightly modifying the SUPG format of [21], they propose a new way to discretize the convection term, which ultimately demonstrates the robustness of the parameters involved in the convergence estimation without requiring sufficiently small mesh sizes.

As we know the application of the virtual element method in the optimal control problem was not reported up to now. Compared with FEM, the computability of the discrete scheme is more important since the virtual element space contains non-polynominal functions. By projection operators and the first-optimize-then-discretize strategy, we construct a computable SUPG-stabilized VEM discrete scheme for the optimal control problem governed by the convection dominated diffusion equation, where the control is implicitly discretized. Moreover, inspired by [23], we use a novel discretization of the convection term that allows us to develop error estimates that are fully robust in the convection dominated cases. We derive an a priori error estimate for the optimal control problem by introducing some auxiliary problems and present a projected gradient algorithm to solve the discrete optimal control problem. Finally, we carry out some numerical examples to verify our theoretical analysis.

The paper is organized as follows. In the next section, we give some preliminary knowledge about virtual element space and the projection operators. In Section 3, the SUPG stabilizing virtual element discrete scheme is constructed. In Section 4, a priori error estimates are derived for the state, adjoint state, and control. In Section 5, we perform some numerical experiments to verify the theoretical results.

Throughout the paper, the symbol ≲ denotes a bound up to a generic positive constant, independent of the mesh size *h*, of the SUPG parameter $\tau_{E}$, of the diffusive coefficient ε and of the transport advective field β. Moreover, the analysis of the three-dimensional case could be developed with very similar arguments.

## 2. Preliminaries

In this section, we introduce the virtual element space and projection operators. Let $T_{h}$ be a family of decompositions of the domain Ω into star-shaped polygonals *E*. $h_{E}$ denotes the diameter of element *E*, i.e., the maximum distance between any two points on element *E* and $h=\underset{E\in T_{h}}{\sup}h_{E}$. We further assume that ∂E denotes the edges of E∈$T_{h}$. $n^{E}$ and $h_{s}$ denote the unit outward normal vector to ∂E and the length of edge *s*, respectively. The following assumption about the mesh is necessary for the theoretical analysis.

**Assumption** **1** ([12]). *We assume the existence of a constant ρ>0 such that, for all h>0 and for all $E\in T_{h}$,*

- *Every element E of $T_{h}$ is star-shaped with respect to a ball of radius bigger or equal to $\rho h_{E}$;*
- *For every element E of $T_{h}$ and every edge s of E, $h_{s}\geq \rho h_{E}$.*

For n=0,1, $P_{n}\left(E\right)$ denotes the space of polynomials of degree ≤n on *E* (with $P_{-\infty }\left(E\right)=\left\{0\right\}$) and the following polynomial projections [12] are given:

- the $L^{2}-projection\;\Pi_{n}^{0}:L^{2}\left(E\right)\to P_{n}\left(E\right)$, defined by
  $$\begin{array}{c} \begin{array}{c} {\left(q,v\right)}_{0,E}={\left(q,\Pi_{n}^{0}v\right)}_{0,E}\;for\;all\;v\in L^{2}\left(E\right)\;and\;q\in P_{n}\left(E\right), \end{array} \end{array}$$
  with an obvious extension for vector functions $\Pi_{n}^{0}:{\left[L^{2}\left(E\right)\right]}^{2}\to {\left[P_{n}\left(E\right)\right]}^{2}$;
- the $H^{1}-projection\;\Pi_{n}^{1}:H^{1}\left(E\right)\to P_{n}\left(E\right)$, defined by
  $$\begin{array}{c} \begin{array}{c} {\left(\nabla q,\nabla v\right)}_{0,E}={\left(\nabla q,\nabla \Pi_{n}^{1}v\right)}_{0,E}\;for\;all\;v\in H^{1}\left(E\right)\;and\;q\in P_{n}\left(E\right) \end{array} \end{array}$$
  plus (to take care of the constant part of $\Pi_{n}^{1}v$):
  $$\begin{array}{c} \begin{array}{c} \int_{\partial E}\left(v-\Pi_{n}^{1}v\right)ds=0. \end{array} \end{array}$$

Then, following [12], the global virtual element space is defined as

$$V_{h}:=\left\{v_{h}\in H_{0}^{1}\left(\Omega \right):v_{h}{|}_{E}\in V_{h}^{E},\forall E\in T_{h}\right\},$$

where

$$\begin{array}{c} \begin{array}{cc} V_{h}^{E} & :=\{v_{h}\in H^{1}\left(E\right)\cap C^{0}\left(\partial E\right):\Delta v_{h}{|}_{E}\in P_{1}\left(E\right),v_{h}{|}_{\partial E}\in P_{1}\left(s\right)\;\forall \;s\in \partial E,\\ & {\left(v_{h},p\right)}_{0,E}={\left(\Pi_{1}^{1}v_{h},p\right)}_{0,E}\;\forall \;p\in P_{1}\left(E\right)/P_{-\infty }\left(E\right)\}, \end{array} \end{array}$$

where the space $P_{1}\left(E\right)/P_{-\infty }\left(E\right)$ denotes the polynomials in $P_{1}\left(E\right)$ that are $L^{2}\left(E\right)$-orthogonal to $P_{-\infty }\left(E\right)$, i.e., the degree of the polynomials in this space are 1 and 0.

Following [12], for each element $E\in T_{h}$, the local virtual element space $V_{h}^{E}$ contains the space $P_{1}\left(E\right)$. The vertices of a polygonal element *E* with $N^{E}$ edges are denoted by $\upsilon_{i}$ for $i=1,\ldots ,N^{E}.$ Because the polygonal element *E* has no internal degrees of freedom, we can simply choose the $\upsilon_{i}$ for $i=1,\ldots ,N^{E}$ to be the degrees of freedom of polygonal element *E*.

**Remark** **1.** *Following [12], from the definition of the virtual element space and the two projection operators, we have $\Pi_{1}^{1}=\Pi_{1}^{0}$.*

**Lemma** **1** ([23]). *Under the Assumption 1, for any $E\in T_{h}$ and for any smooth enough function φ defined on E, it holds*

$$\begin{array}{ccc} \; & ∥\varphi -\Pi_{n}^{0}{\varphi ∥}_{W_{p}^{m}\left(E\right)}≲h_{E}^{s-m}{\left|\varphi \right|}_{W_{p}^{s}\left(E\right)}\;\; & s,m\in N,\;m\leq s\leq n+1,\;p=1,...,\infty ,\\ \; & ∥\varphi -\Pi_{n}^{1}{\varphi ∥}_{m,E}≲h_{E}^{s-m}{\left|\varphi \right|}_{s,E}\;\; & s,m\in N,\;m\leq s\leq n+1,\;s\geq 1,\\ \; & ∥\nabla \varphi -\Pi_{n}^{0}{\nabla \varphi ∥}_{m,E}≲h_{E}^{s-1-m}{\left|\varphi \right|}_{s,E}\;\; & s,m\in N,\;m\leq s\leq n+2,\;s\geq 1. \end{array}$$

## 3. SUPG Stabilizing Virtual Element Approximation for Optimal Control Problem

For all $y,v\in H_{0}^{1}\left(\Omega \right)$, we set

$$\begin{array}{c} \begin{array}{cc} a(y,v) & :=\int_{\Omega }\varepsilon \nabla y\cdot \nabla vd\Omega ,\\ b(y,v) & :=\int_{\Omega }\beta \cdot \nabla yvd\Omega ,\\ c(y,v) & :=\int_{\Omega }\delta yvd\Omega . \end{array} \end{array}$$

By a direct computation, being ∇·β=0, it is easy to see that the bilinear form b(·,·) is skew symmetric, i.e.,

$$b\left(y,v\right)=-b\left(v,y\right)\;for\;ally,v\in H_{0}^{1}\left(\Omega \right).$$

Therefore, we can rewrite the bilinear form b(·,·) as

$$b\left(y,v\right)=\frac{1}{2}\left(b(y,v)-b(v,y)\right).$$

Then, the weak form of optimal control Equations (1) and (2) is characterized by

$$\begin{array}{c} \min_{u\in U_{ad}}J\left(y,u\right):=\frac{1}{2}∥y-y_{d}{∥}^{2}+\frac{\gamma }{2}{∥u∥}^{2} \end{array}$$

subject to

$$\begin{array}{c} \begin{array}{c} A\left(y,v\right)=\left(f+u,v\right)\;\forall v\in H_{0}^{1}\left(\Omega \right), \end{array} \end{array}$$

where

(3) $$\begin{array}{c} \begin{array}{cc} A(y,v) & :=a(y,v)+b(y,v)+c(y,v). \end{array} \end{array}$$

Here, the convection term is rewritten in a skew symmetric form, which is a useful step for the VEM to ensure that the discrete framework preserves the properties of the symmetric and skew-symmetric parts of the bilinear form, see [14,23].

To derive the first order optimality system, we define the Lagrangian functional as follows:

L(y,p,u)=J(y,u)+(f+u,p)−A(y,p).

Taking the directional derivative for L(y,p,u) with respect to *y*, *p*, and *u*, we obtain the continuous first order optimality system of Equations (1) and (2) as follows:

(4) $$\begin{array}{c} \left\{\begin{array}{cc} & A\left(y,w\right)=\left(f+u,w\right),\;\forall w\in H_{0}^{1}\left(\Omega \right),\\ & B\left(p,w\right)=\left(y-y_{d},w\right),\;\forall w\in H_{0}^{1}\left(\Omega \right),\\ & \left(\gamma u+p,v-u\right)\geq 0,\;\forall v\in U_{ad}, \end{array}\right. \end{array}$$

where B(p,w):=a(p,w)−b(p,w)+c(p,w).

Let

$$P_{U_{ad}}\left(u\right)=\max\left\{u_{a},\min\left\{u,u_{b}\right\}\right\}$$

denote the pointwise projection onto the admissible set $U_{ad}$. The optimal inequality is equivalent to $u=P_{U_{ad}}\left(-\frac{1}{\gamma }p\right).$ We can derive that the adjoint state equation has the strong form

(5) $$\begin{array}{c} \left\{\begin{array}{ccc} -\nabla \cdot (\varepsilon \nabla p)-\beta (x)\cdot \nabla p+\delta p & =y-y_{d}\; & \mathrm{in}\;\Omega ,\\ p & =0\; & \mathrm{on}\;\Gamma . \end{array}\right. \end{array}$$

The virtual element discrete scheme of state equation with SUPG stabilization can be defined as follows: find $y_{h}\left(u\right)\in V_{h}$, such that

$$\begin{array}{c} A_{\mathrm{supg},h}\left(y_{h}\left(u\right),w_{h}\right)=\sum_{E\in T_{h}}{\left(\Pi_{1}^{0}\left(f+u\right),w_{h}+\tau_{E}\left(\beta \cdot \Pi_{0}^{0}\nabla w_{h}\right)\right)}_{0,E},\forall w_{h}\in V_{h}. \end{array}$$

Here,

$$\begin{array}{c} \begin{array}{cc} A_{\mathrm{supg},h}\left(v_{h},w_{h}\right) & :=\sum_{E\in T_{h}}A_{\mathrm{supg},h}^{E}\left(v_{h},w_{h}\right):=\sum_{E\in T_{h}}(a_{h}^{E}\left(v_{h},w_{h}\right)+b_{h}^{E}\left(v_{h},w_{h}\right)\\ & \;+c_{h}^{E}\left(v_{h},w_{h}\right)+Q_{h}^{E}\left(v_{h},w_{h}\right)+B_{h}^{E}\left(v_{h},w_{h}\right)+R_{h}^{E}\left(v_{h},w_{h}\right)), \end{array} \end{array}$$

where

$$\begin{array}{cc} \; & a_{h}^{E}\left(v_{h},w_{h}\right):=\int_{E}\varepsilon \Pi_{0}^{0}\nabla v_{h}\cdot \Pi_{0}^{0}\nabla w_{h}dE+\varepsilon S^{E}\left(\left(I-\Pi_{1}^{1}\right)v_{h},\left(I-\Pi_{1}^{1}\right)w_{h}\right),\\ \; & b_{h}^{E}\left(v_{h},w_{h}\right):=\frac{1}{2}[\int_{E}\beta \cdot \nabla \Pi_{1}^{0}v_{h}\Pi_{1}^{0}w_{h}dE-\int_{E}\beta \cdot \nabla \Pi_{1}^{0}w_{h}\Pi_{1}^{0}v_{h}dE\\ \; & \;+\int_{\partial E}\left(\beta \cdot n^{E}\right)\left(I-\Pi_{1}^{0}\right)v_{h}\Pi_{1}^{0}w_{h}ds-\int_{\partial E}\left(\beta \cdot n^{E}\right)\left(I-\Pi_{1}^{0}\right)w_{h}\Pi_{1}^{0}v_{h}ds],\\ \; & c_{h}^{E}\left(v_{h},w_{h}\right):=\int_{E}\delta \Pi_{1}^{0}v_{h}\Pi_{1}^{0}w_{h}dE,\\ \; & Q_{h}^{E}\left(v_{h},w_{h}\right):=\tau_{E}\int_{E}-\varepsilon \nabla \cdot \Pi_{0}^{0}\nabla v_{h}\left(\beta \cdot \Pi_{0}^{0}\nabla w_{h}\right)dE,\\ \; & B_{h}^{E}\left(v_{h},w_{h}\right):=\tau_{E}\int_{E}\beta \cdot \Pi_{0}^{0}\nabla v_{h}\beta \cdot \Pi_{0}^{0}\nabla w_{h}dE+\tau_{E}\beta_{E}^{2}S^{E}\left(\left(I-\Pi_{1}^{1}\right)v_{h},\left(I-\Pi_{1}^{1}\right)w_{h}\right),\\ \; & R_{h}^{E}\left(v_{h},w_{h}\right):=\tau_{E}\int_{E}\delta \Pi_{1}^{0}v_{h}\left(\beta \cdot \Pi_{0}^{0}\nabla w_{h}\right)dE, \end{array}$$

$\tau_{E}>0$ is the SUPG parameter and $\beta_{E}={∥\beta ∥}_{{\left[L^{\infty }\left(E\right)\right]}^{2}}.$ Following [11], $S^{E}$ is any symmetric positive definite bilinear form to be chosen to verify

$$\begin{array}{c} \alpha_{*}|w_{h}{|}_{1,E}^{2}\leq S^{E}\left(w_{h},w_{h}\right)\leq \alpha^{*}{\left|w_{h}\right|}_{1,E}^{2}\;for\;all\;w_{h}\in Ker\left(\Pi_{1}^{1}\right), \end{array}$$

where $\alpha_{*}$ and $\alpha^{*}$ are two positive constants independent of *E* and $h_{E}$.

There are many choices for $S^{E}$, and, following [11], we take the simple choice

$$S^{E}\left(y_{h},w_{h}\right)=\sum_{r=1}^{N^{E}}{\mathrm{dof}}_{r}\left(y_{h}\right){\mathrm{dof}}_{r}\left(w_{h}\right),$$

where ${\mathrm{dof}}_{r}\left(y_{h}\right)$ denotes the value of the *r*th local degree of freedom defining $y_{h}$ in $V_{h}^{E}.$

**Remark** **2.** *Due to limited regularity of the control problem, we restrict k=1 in the virtual element space. In this case, we can observe that $Q_{h}^{E}\left(\cdot ,\cdot \right)=0$ by the definition of the $L^{2}$ projection operator.*

Similar to the state equation, we also use SUPG-stabilized VEM to discretize the adjoint state equation. Then, we can define the discrete first order optimality system as follows: find $\left(u_{h},y_{h},p_{h}\right)\in U_{ad}\times V_{h}\times V_{h}$, such that

(6) $$\begin{array}{c} \left\{\begin{array}{cc} \; & A_{\mathrm{supg},h}\left(y_{h},w_{h}\right)=\sum_{E\in T_{h}}{\left(\Pi_{1}^{0}\left(f+u_{h}\right),w_{h}+\tau_{E}\left(\beta \cdot \Pi_{0}^{0}\nabla w_{h}\right)\right)}_{0,E},\;\forall w_{h}\in V_{h},\\ \; & B_{\mathrm{supg},h}\left(p_{h},w_{h}\right)=\sum_{E\in T_{h}}{\left(\Pi_{1}^{0}\left(y_{h}-y_{d}\right),w_{h}-\tau_{E}\left(\beta \cdot \Pi_{0}^{0}\nabla w_{h}\right)\right)}_{0,E},\;\forall w_{h}\in V_{h},\\ \; & \sum_{E\in T_{h}}{\left(\gamma u_{h}+\Pi_{1}^{0}p_{h},v_{h}-u_{h}\right)}_{0,E}\geq 0,\;\forall v_{h}\in U_{ad}, \end{array}\right. \end{array}$$

where

$$\begin{array}{c} \begin{array}{cc} B_{\mathrm{supg},h}\left(p_{h},w_{h}\right) & :=\sum_{E\in T_{h}}B_{\mathrm{supg},h}^{E}\left(p_{h},w_{h}\right):=\sum_{E\in T_{h}}(a_{h}^{E}\left(p_{h},w_{h}\right)-b_{h}^{E}\left(p_{h},w_{h}\right)\\ & \;+c_{h}^{E}\left(p_{h},w_{h}\right)-Q_{h}^{E}\left(p_{h},w_{h}\right)+B_{h}^{E}\left(p_{h},w_{h}\right)-R_{h}^{E}\left(p_{h},w_{h}\right)). \end{array} \end{array}$$

## 4. A Priori Error Estimates

In this section, we first define the VEM SUPG norm and introduce the auxiliary problems. Then, under certain data assumption, the error estimate of the auxiliary problem and the optimal control problem in the VEM SUPG norm are given. Finally, we derive the error estimate between Equations (4) and (6).

We first define the local VEM SUPG norm

$$\begin{array}{c} \begin{array}{cc} ∥w_{h}{∥}_{\mathrm{supg},E}^{2} & :=\varepsilon ∥\nabla w_{h}{∥}_{0,E}^{2}+\tau_{E}∥\beta \cdot \Pi_{0}^{0}\nabla w_{h}{∥}_{0,E}^{2}+\tau_{E}\beta_{E}^{2}{∥\nabla \left(I-\Pi_{1}^{1}\right)w_{h}∥}_{0,E}^{2}\\ & \;+∥\sqrt{\delta }\left(I-\Pi_{1}^{1}\right)w_{h}{∥}_{0,E}^{2}+{∥\sqrt{\delta }\Pi_{1}^{1}w_{h}∥}_{0,E}^{2} \end{array} \end{array}$$

for all $w_{h}\in H^{1}\left(E\right)$ and the global VEM SUPG norm $∥w_{h}{∥}_{\mathrm{supg}}^{2}:=\sum_{E\in T_{h}}^{}{∥w_{h}∥}_{\mathrm{supg},E}^{2}$ for all $w_{h}\in H^{1}\left(\Omega \right)$. Notice that the norm ${∥\cdot ∥}_{\mathrm{supg},E}$ here is slightly different from the standard SUPG norm ${∥\cdot ∥}_{\mathrm{SUPG},E}$ introduced in standard SUPG theory, i.e.,

$$\begin{array}{c} \begin{array}{c} ∥w_{h}{∥}_{\mathrm{SUPG},E}^{2}:=\varepsilon ∥\nabla w_{h}{∥}_{0,E}^{2}+\tau_{E}∥\beta \cdot \nabla w_{h}{∥}_{0,E}^{2}+{∥\sqrt{\delta }w_{h}∥}_{0,E}^{2}. \end{array} \end{array}$$

However, for all $w_{h}\in H^{1}\left(E\right)$, using the fact that

$$\begin{array}{c} \begin{array}{c} ∥\nabla p_{h}-\Pi_{0}^{0}\nabla p_{h}{∥}_{0,E}=∥\left(I-\Pi_{0}^{0}\right)\left(\nabla p_{h}-\nabla \Pi_{1}^{1}p_{h}\right){∥}_{0,E}\leq {∥\nabla p_{h}-\nabla \Pi_{1}^{1}p_{h}∥}_{0,E} \end{array} \end{array}$$

we arrive at

$$\begin{array}{c} \begin{array}{cc} ∥\beta \cdot \nabla w_{h}{∥}_{0,E}^{2} & =∥\beta \cdot \left(\nabla w_{h}-\Pi_{0}^{0}\nabla w_{h}+\Pi_{0}^{0}\nabla w_{h}\right){∥}_{0,E}^{2}\\ & \leq 2∥\beta \cdot \Pi_{0}^{0}\nabla w_{h}{∥}_{0,E}^{2}+2\beta_{E}^{2}{∥\left(I-\Pi_{0}^{0}\right)\nabla w_{h}∥}_{0,E}^{2}\\ & \leq 2∥\beta \cdot \Pi_{0}^{0}\nabla w_{h}{∥}_{0,E}^{2}+2\beta_{E}^{2}{∥\nabla \left(I-\Pi_{1}^{1}\right)w_{h}∥}_{0,E}^{2} \end{array} \end{array}$$

and

$$\begin{array}{c} \begin{array}{cc} ∥\sqrt{\delta }w_{h}{∥}_{0,E}^{2} & \leq ∥\sqrt{\delta }\left(w_{h}-\Pi_{1}^{1}w_{h}+\Pi_{1}^{1}w_{h}\right){∥}_{0,E}^{2}\leq 2∥\sqrt{\delta }\Pi_{1}^{1}w_{h}{∥}_{0,E}^{2}+2{∥\sqrt{\delta }\left(I-\Pi_{1}^{1}\right)w_{h}∥}_{0,E}^{2}. \end{array} \end{array}$$

This implies that the standard SUPG norm can be controlled by the VEM SUPG norm.

**Lemma** **2** ([23]). *Under the Assumption 1, if there exists a constant $C_{\tau }\in \left(0,2\right)$ such that the parameters $\tau_{E}$ satisfy $\tau_{E}\leq \frac{C_{\tau }}{\delta },\;\forall E\in T_{h},$ the bilinear form $A_{\mathrm{supg},h}^{E}\left(\cdot ,\cdot \right)$ and $B_{\mathrm{supg},h}^{E}\left(\cdot ,\cdot \right)$ satisfy for all $w_{h}\in V_{h}\left(E\right)$ the coercivity inequality*

(7) $$\begin{array}{c} \begin{array}{ccc} ∥w_{h}{∥}_{\mathrm{supg},E}^{2} & ≲A_{\mathrm{supg},h}^{E}\left(w_{h},w_{h}\right),\;{∥w_{h}∥}_{\mathrm{supg},E}^{2} & ≲B_{\mathrm{supg},h}^{E}\left(w_{h},w_{h}\right). \end{array} \end{array}$$

To derive an a priori error estimate, we need to introduce the following auxiliary problems:

(8) $$\begin{array}{c} \left\{\begin{array}{cc} & A\left(y\left(u_{h}\right),w\right)=\left(f+u_{h},w\right),\;\forall w\in H_{0}^{1}\left(\Omega \right),\\ & B\left(p\left(y_{h}\right),w\right)=\left(y_{h}-y_{d},w\right),\;\forall w\in H_{0}^{1}\left(\Omega \right),\\ & B\left(p\left(u_{h}\right),w\right)=\left(y\left(u_{h}\right)-y_{d},w\right),\;\forall w\in H_{0}^{1}\left(\Omega \right) \end{array}\right. \end{array}$$

and

(9) $$\begin{array}{c} \left\{\begin{array}{cc} & A_{\mathrm{supg},h}\left(y_{h}\left(u\right),w_{h}\right)=\sum_{E\in T_{h}}{\left(\Pi_{1}^{0}\left(f+u\right),w_{h}+\tau_{E}\left(\beta \cdot \Pi_{0}^{0}\nabla w_{h}\right)\right)}_{0,E},\;\forall w_{h}\in V_{h},\\ & B_{\mathrm{supg},h}\left(p_{h}\left(u\right),w_{h}\right)=\sum_{E\in T_{h}}{\left(\Pi_{1}^{0}\left(y_{h}\left(u\right)-y_{d}\right),w_{h}-\tau_{E}\left(\beta \cdot \Pi_{0}^{0}\nabla w_{h}\right)\right)}_{0,E},\;\forall w_{h}\in V_{h},\\ & B_{\mathrm{supg},h}\left(p_{h}\left(y\right),w_{h}\right)=\sum_{E\in T_{h}}{\left(\Pi_{1}^{0}\left(y-y_{d}\right),w_{h}-\tau_{E}\left(\beta \cdot \Pi_{0}^{0}\nabla w_{h}\right)\right)}_{0,E},\;\forall w_{h}\in V_{h}. \end{array}\right. \end{array}$$

**Assumption** **2** (Data assumption). *The solutions y,p,u of the optimal control problem and the $f,y_{d}$ satisfy:*

$$f,u,y_{d}\in H^{1}\left(T_{h}\right),\;y,p\in H^{2}\left(T_{h}\right).$$

Note that $\left(y_{h}\left(u\right),p_{h}\left(y\right)\right)$ are SUPG VEM approximation of (y,p). The following results are not restrictive to $\beta_{E}>0$ since $\beta_{E}=0$ implies ${\beta |}_{E}=0$ and thus the corresponding terms vanish.

**Lemma** **3** ([23]). *Let (y,p) and $\left(y_{h}\left(u\right),p_{h}\left(y\right)\right)$ be the solutions of (4) and (9), respectively. Then, the following estimates hold under the Assumptions 1, 2 and, in the case of a convection dominated regime, i.e., $\tau_{E}=\min\left\{\frac{h_{E}}{\beta_{E}},\frac{h_{E}^{2}}{\varepsilon },\frac{C_{\tau }}{\delta }\right\}=\frac{h_{E}}{\beta_{E}}$*

$$\begin{array}{c} \begin{array}{cc} ∥y-y_{h}{\left(u\right)∥}_{\mathrm{supg}}^{2}+{∥p-p_{h}\left(y\right)∥}_{\mathrm{supg}}^{2} & ≲\sum_{E\in T_{h}}\left(h_{E}^{3}\left(\beta_{E}+\beta_{E}^{-1}\right)+\varepsilon h_{E}^{2}+\beta_{E}^{-1}\varepsilon^{2}h_{E}+h_{E}^{4}+\beta_{E}^{-1}h_{E}^{5}\right)\\ & ≲\sum_{E\in T_{h}}\left(h_{E}^{3}\left(\beta_{E}+\beta_{E}^{-1}+h_{E}+\beta_{E}^{-1}h_{E}^{2}\right)\right)\\ & =O(h^{3}). \end{array} \end{array}$$

Next, we derive a priori error estimates for SUPG VEM approximation of the optimal control problem.

**Theorem** **1.** *Let (y,p,u) and $\left(y_{h},p_{h},u_{h}\right)$ be the solutions of (4) and (6), respectively. Then, we have that*

$$\begin{array}{c} \begin{array}{c} ∥u-u_{h}{∥}_{0,\Omega }≲h^{2}+{∥p_{h}-p\left(u_{h}\right)∥}_{0,\Omega }. \end{array} \end{array}$$

**Proof.**  Define

$${\hat{J}}^{\prime }\left(u\right)\left(v-u\right)=\int_{\Omega }\left(\gamma u+p\left(u\right)\right)\left(v-u\right)dx.$$

Note that

$$\begin{array}{c} \begin{array}{c} {\hat{J}}^{\prime }\left(u\right)\left(u-u_{h}\right)-{\hat{J}}^{\prime }\left(u_{h}\right)\left(u-u_{h}\right)=\gamma \int_{\Omega }{\left(u-u_{h}\right)}^{2}dx+\int_{\Omega }\left(p\left(u\right)-p\left(u_{h}\right)\right)\left(u-u_{h}\right)dx. \end{array} \end{array}$$

By the auxiliary Equation (8), we deduce

$$\begin{array}{c} \begin{array}{cc} \int_{\Omega }\left(p\left(u\right)-p\left(u_{h}\right)\right)\left(u-u_{h}\right)dx & =A(y\left(u\right)-y\left(u_{h}\right),p\left(u\right)-p\left(u_{h}\right))\\ & =B(p\left(u\right)-p\left(u_{h}\right),y\left(u\right)-y\left(u_{h}\right))\\ & ={\left(y\left(u\right)-y\left(u_{h}\right),y\left(u\right)-y\left(u_{h}\right)\right)}_{0,\Omega }\geq 0. \end{array} \end{array}$$

Then, we have

$$\begin{array}{c} \begin{array}{cc} \gamma ∥u-u_{h}{∥}_{0,\Omega }^{2} & \leq {\hat{J}}^{\prime }\left(u\right)\left(u-u_{h}\right)-{\hat{J}}^{\prime }\left(u_{h}\right)\left(u-u_{h}\right)=\int_{\Omega }\left(\gamma u+p-\gamma u_{h}-p\left(u_{h}\right)\right)\left(u-u_{h}\right)dx\\ & ={\left(\gamma u+p,u-u_{h}\right)}_{0,\Omega }+\sum_{E\in T_{h}}{\left(\gamma u_{h}+\Pi_{1}^{0}p_{h},u_{h}-u\right)}_{0,E}\\ & \;+\sum_{E\in T_{h}}{\left(\Pi_{1}^{0}p_{h}-p\left(u_{h}\right),u-u_{h}\right)}_{0,E}\\ & \leq 0+0+\sum_{E\in T_{h}}{\left(\Pi_{1}^{0}p_{h}-p\left(u_{h}\right),u-u_{h}\right)}_{0,E}. \end{array} \end{array}$$

This shows

$$∥u-u_{h}{∥}_{0,\Omega }≲{\left(\sum_{E\in T_{h}}{∥\Pi_{1}^{0}p_{h}-p\left(u_{h}\right)∥}_{0,E}^{2}\right)}^{\frac{1}{2}}.$$

Note that

$$\begin{array}{c} \begin{array}{cc} ∥\Pi_{1}^{0}p_{h}-p\left(u_{h}\right){∥}_{0,E} & \leq \;∥\Pi_{1}^{0}p_{h}-\Pi_{1}^{0}p\left(u_{h}\right){∥}_{0,E}+{∥\Pi_{1}^{0}p\left(u_{h}\right)-p\left(u_{h}\right)∥}_{0,E}\\ & \leq ∥p_{h}-p\left(u_{h}\right){∥}_{0,E}+{∥\Pi_{1}^{0}p\left(u_{h}\right)-p\left(u_{h}\right)∥}_{0,E}. \end{array} \end{array}$$

Then, by Lemma 1, we have

$$∥u-u_{h}{∥}_{0,\Omega }≲h^{2}+{∥p_{h}-p\left(u_{h}\right)∥}_{0,\Omega }.$$

   □

**Theorem** **2.** *Under the Assumptions 1 and 2 and, in case of a convection dominated regime, let (y,p,u) and $\left(y_{h},p_{h},u_{h}\right)$ be the solutions of Equations (4) and (6), respectively. Then, we have following estimate:*

$$\begin{array}{c} \begin{array}{c} ∥y-y_{h}{∥}_{\mathrm{supg}}+∥p-p_{h}{∥}_{\mathrm{supg}}+{∥u-u_{h}∥}_{0,\Omega }≲h^{3/2}. \end{array} \end{array}$$

**Proof.**  We decompose the errors $y-y_{h}$ and $p-p_{h}$ into

$$y-y_{h}=y-y_{h}\left(u\right)+y_{h}\left(u\right)-y_{h},\;\;p-p_{h}=p-p_{h}\left(u\right)+p_{h}\left(u\right)-p_{h}.$$

By the Lemma 3, we have

$$∥y-y_{h}{\left(u\right)∥}_{\mathrm{supg}}≲h^{3/2}.$$

Moreover, by the governing equation of $y_{h}$, $y_{h}\left(u\right)$ in (6) and (9), respectively, we have

$$A_{\mathrm{supg},h}\left(y_{h}\left(u\right)-y_{h},w_{h}\right)=\sum_{E\in T_{h}}{\left(\Pi_{1}^{0}\left(u-u_{h}\right),w_{h}+\tau_{E}\beta \cdot \Pi_{0}^{0}\nabla w_{h}\right)}_{0,E}.$$

Let $w_{h}=y_{h}\left(u\right)-y_{h}.$ Recalling the coercivity of $A_{\mathrm{supg},h}$ in (7), $\Pi_{1}^{0}=\Pi_{1}^{1}$ and the fact that $\tau_{E}\leq \frac{C_{\tau }}{\delta }$ we can get

(10) $$\begin{array}{cc} \; & ∥y_{h}\left(u\right)-y_{h}{∥}_{\mathrm{supg}}^{2}≲A_{\mathrm{supg},h}\left(y_{h}\left(u\right)-y_{h},y_{h}\left(u\right)-y_{h}\right)\\ \; & =\sum_{E\in T_{h}}{\left(\Pi_{1}^{0}\left(u-u_{h}\right),y_{h}\left(u\right)-y_{h}+\tau_{E}\beta \cdot \Pi_{0}^{0}\nabla \left(y_{h}\left(u\right)-y_{h}\right)\right)}_{0,E}\\ \; & \leq ∥u-u_{h}{∥}_{0,\Omega }(\sum_{E\in T_{h}}∥\Pi_{1}^{0}\left(y_{h}\left(u\right)-y_{h}\right){{∥}_{0,E}^{2})}^{1/2}\\ \; & \;+∥u-u_{h}{∥}_{0,\Omega }(\sum_{E\in T_{h}}∥\tau_{E}\beta \cdot \Pi_{0}^{0}\nabla \left(y_{h}\left(u\right)-y_{h}\right){{∥}_{0,E}^{2})}^{1/2}\\ \; & ≲∥u-u_{h}{∥}_{0,\Omega }∥y_{h}\left(u\right)-y_{h}{∥}_{\mathrm{supg}}+∥u-u_{h}{∥}_{0,\Omega }(\sum_{E\in T_{h}}\tau_{E}∥y_{h}\left(u\right)-y_{h}{{∥}_{\mathrm{supg},E}^{2})}^{1/2}\\ \; & ≲∥u-u_{h}{∥}_{0,\Omega }∥y_{h}\left(u\right)-y_{h}{∥}_{\mathrm{supg}}+∥u-u_{h}{∥}_{0,\Omega }(\sum_{E\in T_{h}}\frac{C_{\tau }}{\delta }∥y_{h}\left(u\right)-y_{h}{{∥}_{\mathrm{supg},E}^{2})}^{1/2}\\ \; & ≲∥u-u_{h}{∥}_{0,\Omega }{∥y_{h}\left(u\right)-y_{h}∥}_{\mathrm{supg}}. \end{array}$$

This implies

$$∥y_{h}\left(u\right)-y_{h}{∥}_{\mathrm{supg}}≲{∥u-u_{h}∥}_{0,\Omega }.$$

Combining the above inequalities gives

$$∥y-y_{h}{∥}_{\mathrm{supg}}≲h^{3/2}+{∥u-u_{h}∥}_{0,\Omega }.$$

By Lemma 3, we also have

$$∥p-p_{h}{\left(y\right)∥}_{\mathrm{supg}}≲h^{3/2}.$$

Similar to the estimate (10), by the definition of $p_{h}\left(y\right)$, $p_{h}\left(u\right)$ in (9), we also derive

$$\begin{array}{c} \begin{array}{c} ∥p_{h}\left(y\right)-p_{h}{\left(u\right)∥}_{\mathrm{supg}}≲{∥y-y_{h}\left(u\right)∥}_{\mathrm{supg}}≲h^{3/2} \end{array} \end{array}$$

and

$$\begin{array}{c} \begin{array}{c} ∥p_{h}\left(u\right)-p_{h}{∥}_{\mathrm{supg}}≲∥y_{h}\left(u\right)-y_{h}{∥}_{\mathrm{supg}}≲{∥u-u_{h}∥}_{0,\Omega }. \end{array} \end{array}$$

This implies

$$\begin{array}{c} \begin{array}{c} ∥p-p_{h}{∥}_{\mathrm{supg}}≲h^{3/2}+{∥u-u_{h}∥}_{0,\Omega }. \end{array} \end{array}$$

From the coercivity of *B* and definition of $p(y_{h})$, $p(u_{h})$ in (8), we can deduce

$$\begin{array}{c} \begin{array}{cc} ∥p\left(y_{h}\right)-p\left(u_{h}\right){∥}_{0,\Omega }^{2} & ≲B(p\left(y_{h}\right)-p\left(u_{h}\right),p\left(y_{h}\right)-p\left(u_{h}\right))\\ & ={\left(y_{h}-y\left(u_{h}\right),p\left(y_{h}\right)-p\left(u_{h}\right)\right)}_{0,\Omega }\\ & \leq ∥y_{h}-y\left(u_{h}\right){∥}_{0,\Omega }{∥p\left(y_{h}\right)-p\left(u_{h}\right)∥}_{0,\Omega }. \end{array} \end{array}$$

Thus, we can get $∥p\left(y_{h}\right)-p\left(u_{h}\right){∥}_{0,\Omega }\leq {∥y_{h}-y\left(u_{h}\right)∥}_{0,\Omega }$. Using the results of [23], we  obtain

$$\begin{array}{c} \begin{array}{c} ∥p_{h}-p\left(y_{h}\right){∥}_{\mathrm{supg}}≲h^{3/2},\;{∥y_{h}-y\left(u_{h}\right)∥}_{\mathrm{supg}}≲h^{3/2}. \end{array} \end{array}$$

By the triangle inequality and the relationship between ${∥\cdot ∥}_{\mathrm{SUPG}}$ and ${∥\cdot ∥}_{\mathrm{supg}}$, we arrive at

(11) $$\begin{array}{c} \begin{array}{cc} ∥p_{h}-p\left(u_{h}\right){∥}_{0,\Omega } & \leq ∥p_{h}-p\left(y_{h}\right){∥}_{0,\Omega }+{∥p\left(y_{h}\right)-p\left(u_{h}\right)∥}_{0,\Omega }\\ & ≲∥p_{h}-p\left(y_{h}\right){∥}_{\mathrm{SUPG}}+{∥y_{h}-y\left(u_{h}\right)∥}_{\mathrm{SUPG}}\\ & ≲∥p_{h}-p\left(y_{h}\right){∥}_{\mathrm{supg}}+{∥y_{h}-y\left(u_{h}\right)∥}_{\mathrm{supg}}≲h^{3/2}. \end{array} \end{array}$$

From Theorem 1 and Equation (11), we have the following estimate:

$$∥u-u_{h}{∥}_{0,\Omega }≲h^{3/2}+h^{2}.$$

Inserting the above estimate into the estimates of state and adjoint state yields the final result.    □

## 5. Numerical Results

In this section, we give the mesh types of our numerical experiments and introduce a projected gradient algorithm based on the SUPG stabilized discrete first order optimality system (6) to verify our a priori analysis. Since the VEM solutions $y_{h}$ and $p_{h}$ are not explicitly known inside the elements, we use $e_{y,0},e_{y,1},c_{y}$ to represent the $L^{2}$ norm, $H^{1}$ norm, and standard SUPG norm between *y* and $\Pi_{1}^{1}y_{h}$. Similarly, $e_{p,0},e_{p,1},c_{p}$ denote the $L^{2}$ norm, $H^{1}$ norm and standard SUPG norm between *p* and $\Pi_{1}^{1}p_{h}$, and $e_{u,0}$ denotes the $L^{2}$ norm between *u* and $u_{h}$.

**Figure 1:**
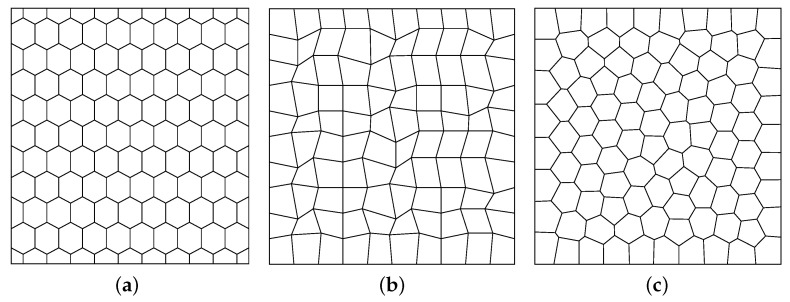
Three mesh types. (**a**) Hexagonal mesh; (**b**) Distorted square mesh; (**c**) Lloyd mesh.

For the mesh types, we consider distorted square, hexagonal, and Lloyd mesh ([24]), which are shown as Figure 1a, Figure 1b, Figure 1c in Figure 1, respectively.

The projected gradient algorithm is given below (Algorithm 1):
**Algorithm 1:** Projected gradient algorithm.Require:
      Regularization parameter γ and tolerance error η. Ensure:
Choose the initial value $u_{h}$. Set error=1.While error>η doSolving the state equation in the discrete first order optimality system (6) to get state variable  $y_{h}$;Solving the adjoint state equation in the discrete first order optimality system (6) to obtain adjoint state variable $p_{h}$;Control variable $u_{hnew}$ are obtained by using the projection $P_{U_{ad}}$;
$u_{hnew}{|}_{E}=P_{U_{ad}}\left(-\frac{1}{\gamma }\Pi_{1}^{0}\left(p_{h}{|}_{E}\right)\right),\forall E\in T_{h}.$Calculate the error:
$$error=∥u_{h}-u_{hnew}{∥}_{L^{2}}.$$Update control variable $u_{h}=u_{hnew};$end while

For the detailed calculation process of the projection and the influence of projection on the convergence rate, we can refer to the literature [14].

**Example** **1.** *Consider the optimal control Equations (1) and (2) on the unit square Ω=[0,1]×[0,1]. Let $u_{a}=-0.3$, $u_{b}=0$, γ=1, and*

$$\varepsilon =10^{-10},\;\beta =\left[\begin{array}{c} \frac{1}{3}+10y{\left(x+y^{2}\right)}^{4}\\ -\frac{1}{2}-5{\left(x+y^{2}\right)}^{4} \end{array}\right],\;\delta =1.$$

*The exact solutions are chosen to be*

$$\begin{array}{c} \begin{array}{cc} y(x_{1},x_{2}) & =200x_{1}x_{2}\left(1-x_{1}\right)\left(1-x_{2}\right)\left(x_{1}-3/5\right)\left(x_{2}-3/5\right),\\ p(x_{1},x_{2}) & =80x_{1}x_{2}\left(1-x_{1}\right)\left(1-x_{2}\right)\left(x_{1}-3/5\right)\left(x_{2}-3/5\right),\\ u(x_{1},x_{2}) & =P_{U_{ad}}\left(-p\right). \end{array} \end{array}$$

*f and $y_{d}$ can be determined from the exact solutions y,p,u.*

We choose $C_{\tau }=0.8$ and let $r_{1}=\max_{E\in T_{h}}\frac{h_{E}}{\beta_{E}}$, $r_{2}=\min_{E\in T_{h}}\frac{h_{E}^{2}}{\varepsilon }$, $r_{3}=\frac{C_{\tau }}{\delta }$ under a fixed mesh. In order to ensure the dominance of the convection term, i.e., the choice of parameter $\tau_{E}$ is $\frac{h_{E}}{\beta_{E}}$, numerical comparisons of $r_{1}$, $r_{2}$, and $r_{3}$ are shown in Table 1 and Table 2 for each type of grid subdivision with $\varepsilon =10^{-10}$. We can see that the convection term is always dominant. We also give the convergence rates of the above norms for distorted square, hexagonal, and Lloyd mesh with the SUPG term. We can observe that the convergence orders of $L^{2}$ errors, $H^{1}$ errors, and standard convective norm errors approximate 2, 1, and 1.5, respectively, which are the optimal convergence rates. The experimental data verify our theoretical analysis.

**Figure 2:**
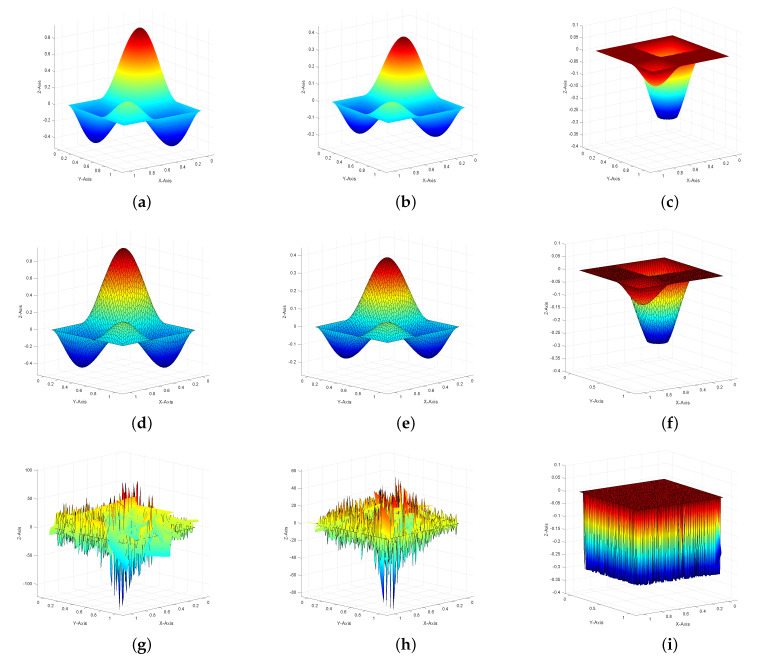
The profiles of the exact solutions of state (**a**), adjoint state (**b**), control (**c**), SUPG-stabilized discretized optimal state $y_{h}$ (**d**), adjoint state $p_{h}$ (**e**), and control $u_{h}$ (**f**). (**g**–**i**) are the profiles of the unstabilized numerically computed state, adjoint state, and control for Example 1 on Lloyd mesh with $\varepsilon =10^{-10}$.

In Figure 2a–c, we plot the profiles of the exact solutions of state, adjoint state, and control with $\varepsilon =10^{-10}$, respectively. Figure 2 shows an intuitive comparison between the unstabilized numerically computed state, adjoint state, control and the numerically computed solutions obtained using the SUPG stabilization on Lloyd mesh with $\varepsilon =10^{-10}$. By comparison, we can find that the numerical solutions Figure 2d–f show a very good agreement with the exact solutions and show a good stability as ε→0 when the SUPG term exists. The quality of the discrete solutions deteriorates obviously when there is no SUPG term (Figure 2g–i).

**Example** **2.** *We consider the Equations (1) and (2) with a modified objective functional*

$$\begin{array}{c} J\left(y,u\right):=\frac{1}{2}∥y-y_{d}{∥}^{2}+\frac{\gamma }{2}{∥u-u_{0}∥}^{2} \end{array}$$

*and $\varepsilon =10^{-4},\beta =\left(1,0\right),\delta =1,\gamma =1$ on the unit square Ω=[0,1]×[0,1]. The exact solution of the optimal control problem is as follows:*

$$\begin{array}{c} \begin{array}{cc} y & =4e^{\left(-\left({\left(x_{1}-0.7\right)}^{2}+{\left(x_{2}-0.7\right)}^{2}\right)/\sqrt{\varepsilon }\right)}sin\left(\pi x_{1}\right)sin\left(\pi x_{2}\right),\\ p & =e^{\left(-\left({\left(x_{1}-0.7\right)}^{2}+{\left(x_{2}-0.7\right)}^{2}\right)/\sqrt{\varepsilon }\right)}sin\left(\pi x_{1}\right)sin\left(\pi x_{2}\right),\\ u & =\max\{0,cos\left(\pi x_{1}\right)cos\left(\pi x_{2}\right)-1\}. \end{array} \end{array}$$

*These functions are inserted into the equations and the corresponding source terms f, $y_{0}$, and $u_{0}$ are computed.*

**Figure 3:**
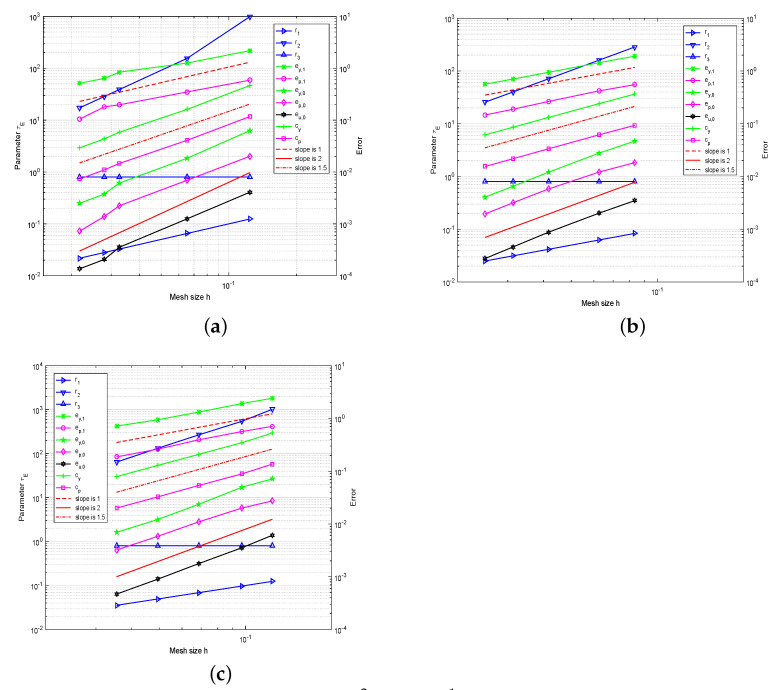
The convergence history of the $L^{2}$ errors, $H^{1}$ errors, standard SUPG norm errors, and the choice of parameter $\tau_{E}$ on distorted square meshes (**a**), hexagonal meshes (**b**), and Lloyd meshes (**c**) with SUPG term for Example 2.

We also choose $C_{\tau }=0.8$ and let $r_{1}=\max_{E\in T_{h}}\frac{h_{E}}{\beta_{E}}$, $r_{2}=\min_{E\in T_{h}}\frac{h_{E}^{2}}{\varepsilon }$, $r_{3}=\frac{C_{\tau }}{\delta }$ under a fixed mesh. In Figure 3, we show the convergence graphs for three meshes with an SUPG term under the above norms and the intuitive comparison of $r_{1}$, $r_{2}$ and $r_{3}$, respectively. We can observe that the convergence orders of $L^{2}$ errors, $H^{1}$ errors and standard SUPG norm errors are approximately parallel to the lines with slopes 2, 1, and 1.5 in the convection dominated regime. We can observe that the convergence rates are in agreement with the theoretical prediction.

**Figure 4:**
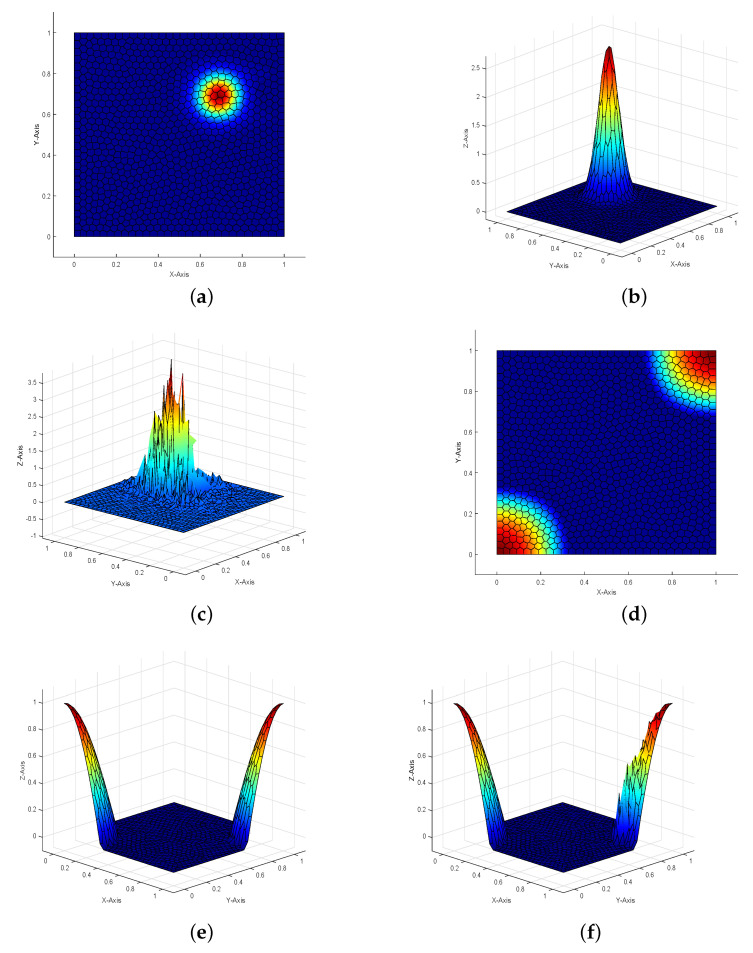
The contour-lines and profiles of the SUPG-stabilized VEM discretized optimal state $y_{h}$, control $u_{h}$, and unstabilized state $y_{h}$, control $u_{h}$ for Example 2 on Lloyd mesh. (**a**) The contour-line of stabilised state; (**b**) The profile of stabilised state; (**c**) The profile of unstabilised state; (**d**) The contour-line of stabilised control; (**e**) The profile of stabilised control; (**f**) The profile of unstabilised control.

The contour-lines and profiles of the stabilized numerically computed state, control, and the profiles of the numerically computed state and control without SUPG term on Lloyd mesh are shown in Figure 4, respectively. We can observe that the quality of the numerical solutions are good when the SUPG term is present; otherwise, the numerical solutions are obviously destroyed, which implies that the SUPG term has a good effect.

**Example** **3.** *In this example, we set $\Omega ={\left[0,1\right]}^{2},\beta ={\left(2,3\right)}^{T},\delta =1,\gamma =1,$ and $\varepsilon =10^{-4}$. The exact solutions are given by*

$$\begin{array}{c} \begin{array}{cc} y & =\frac{1}{1+e^{-\frac{\sqrt{{\left(x_{1}-1\right)}^{2}+{\left(x_{2}-1\right)}^{2}}-0.7}{\sqrt{\varepsilon }}}},\\ p & =\frac{1}{1+e^{-\frac{\sqrt{x_{1}^{2}+x_{2}^{2}}-0.5}{\sqrt{\varepsilon }}}},\\ u & =\max\{-0.8,-p\}. \end{array} \end{array}$$

*The right-hand term f and the desired state $y_{d}$ can be calculated by the exact solutions and governing equations.*

**Figure 5:**
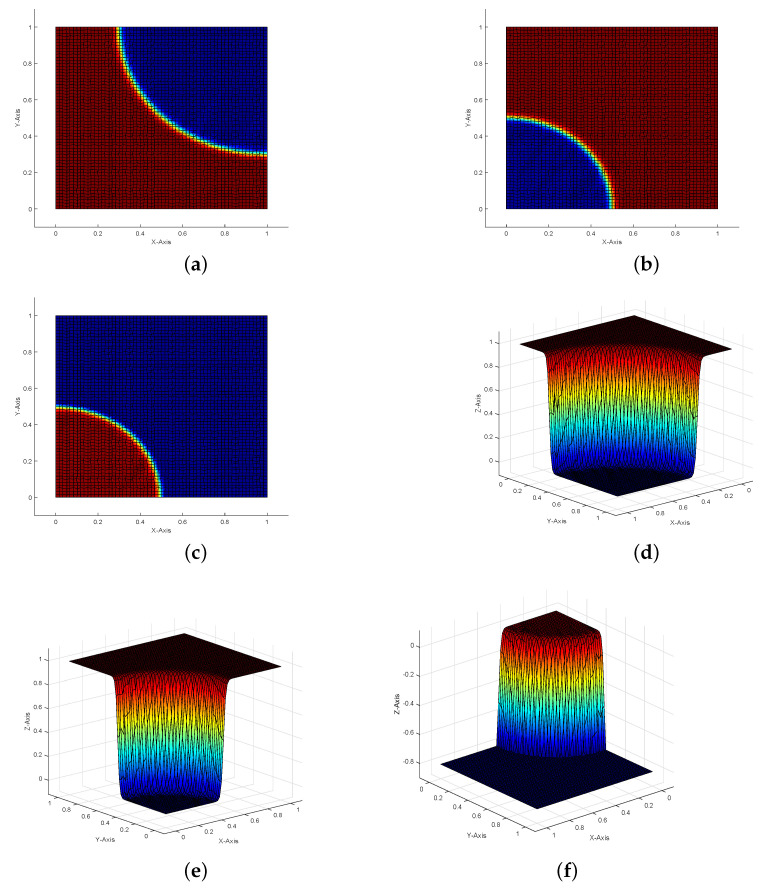
The contour-lines and profiles of the SUPG-stabilized VEM discretized optimal state $y_{h}$, adjoint state $p_{h}$, and control $u_{h}$ for Example 3 on distorted square mesh. (**a**) The contour-line of stabilised state; (**b**) The contour-line of stabilised adjoint state; (**c**) The contour-line of stabilised control; (**d**) The profile of stabilised state; (**e**) The profile of stabilised adjoint state; (**f**) The profile of stabilised control.

**Figure 6:**
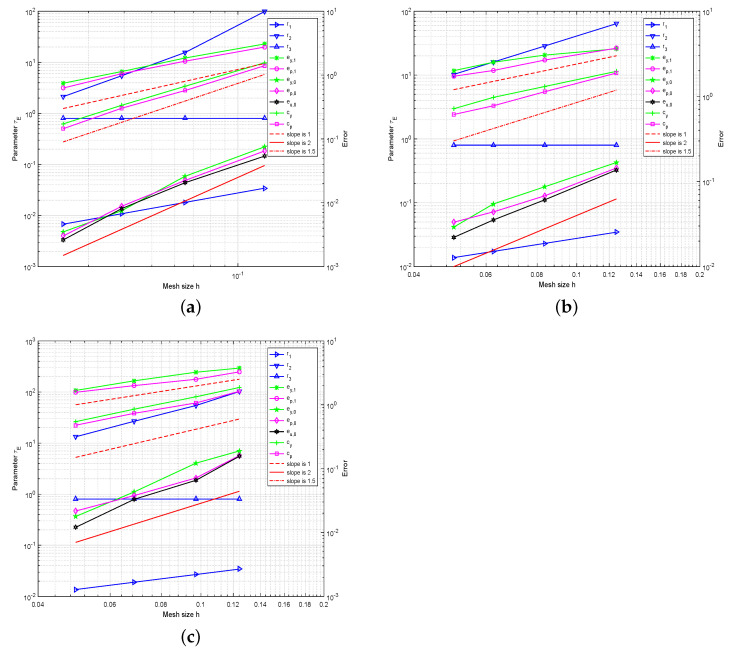
The convergence history of the $L^{2}$ errors, $H^{1}$ errors, standard SUPG norm errors, and the choice of parameter $\tau_{E}$ on distorted square meshes (**a**), hexagonal meshes (**b**), and Lloyd meshes (**c**) with the SUPG term for Example 3.

In this example, the state *y* and adjoint state *p* have sharp interior layers along the circles ${\left(x_{1}-1\right)}^{2}+{\left(x_{2}-1\right)}^{2}=0.7^{2}$ and $x_{1}^{2}+x_{2}^{2}=0.5^{2}$, respectively. Figure 5 shows the contour-lines and profiles of stabilized numerical solutions on the distorted square mesh and the quality of the numerical solutions are not destroyed by the numerical oscillation. We can see that our method represents and processes the interior layers well. The corresponding convergence rates are given in Figure 6, which are also consistent with our theoretical analysis.

## 6. Conclusions

In this paper, we attempt to apply SUPG-stabilized VEM to approximate an optimal control problem governed by a convection dominated diffusion equation with pointwise control constraint. A priori error estimates are derived. The theoretical findings are verified by numerical examples.

Since the VEM has great flexibility in the mesh partition, in our future work, we are going to investigate VEM approximation of an optimal control problem governed by fractional advection-diffusion–reaction equations ([25,26,27]).

## Figures and Tables

**Table 1:** Example 1: Errors and convergence rates of *y* and *p* in standard convective norm on three meshes with $\varepsilon =10^{-10}$.

Mesh	*h*	$r_{1}$	$r_{2}$	$r_{3}$	$c_{y}$	Rate	$c_{p}$	Rate
Distorted square mesh	0.131	0.193	$6.225\times 10^{7}$	0.800	$3.097\times 10^{-1}$		$1.237\times 10^{-1}$	
	0.098	0.153	$3.412\times 10^{7}$	0.800	$2.049\times 10^{-1}$	1.44	$8.197\times 10^{-2}$	1.43
	0.049	0.081	$8.531\times 10^{6}$	0.800	$7.737\times 10^{-2}$	1.41	$3.088\times 10^{-2}$	1.41
	0.033	0.054	$3.892\times 10^{6}$	0.800	$4.114\times 10^{-2}$	1.56	$1.641\times 10^{-2}$	1.56
	0.022	0.036	$1.729\times 10^{6}$	0.800	$2.119\times 10^{-2}$	1.64	$8.424\times 10^{-3}$	1.64
Hexagonal mesh	0.125	0.208	$6.406\times 10^{7}$	0.800	$3.852\times 10^{-1}$		$1.522\times 10^{-1}$	
	0.063	0.104	$1.602\times 10^{7}$	0.800	$1.533\times 10^{-1}$	1.33	$6.200\times 10^{-2}$	1.30
	0.042	0.069	$7.118\times 10^{6}$	0.800	$8.804\times 10^{-2}$	1.37	$3.557\times 10^{-2}$	1.37
	0.036	0.059	$5.230\times 10^{6}$	0.800	$7.107\times 10^{-2}$	1.40	$2.864\times 10^{-2}$	1.40
	0.031	0.052	$3.996\times 10^{6}$	0.800	$5.894\times 10^{-2}$	1.41	$2.375\times 10^{-2}$	1.41
Lloyd mesh	0.201	0.274	$2.136\times 10^{8}$	0.800	$7.215\times 10^{-1}$		$2.954\times 10^{-1}$	
	0.097	0.158	$5.442\times 10^{7}$	0.800	$2.539\times 10^{-1}$	1.44	$1.005\times 10^{-1}$	1.49
	0.070	0.116	$2.603\times 10^{7}$	0.800	$1.507\times 10^{-1}$	1.55	$6.047\times 10^{-2}$	1.51
	0.049	0.073	$1.274\times 10^{7}$	0.800	$9.224\times 10^{-2}$	1.41	$3.691\times 10^{-2}$	1.42
	0.035	0.052	$6.429\times 10^{6}$	0.800	$5.382\times 10^{-2}$	1.66	$2.145\times 10^{-2}$	1.67

**Table 2:** Example 1: Errors and convergence rates of y,p and *u* in $L^{2}$, $H^{1}$ norms and $L^{2}$ norm, respectively, on three meshes with $\varepsilon =10^{-10}$.

Mesh	$e_{y,0}$	Rate	$e_{y,1}$	Rate	$e_{p,1}$	Rate	$e_{p,0}$	Rate	$e_{u,0}$	Rate
Distorted square mesh	$3.425\times 10^{-2}$		$5.752\times 10^{-1}$		$2.277\times 10^{-1}$		$1.355\times 10^{-2}$		$8.982\times 10^{-3}$	
	$1.952\times 10^{-2}$	1.95	$4.289\times 10^{-1}$	1.02	$1.670\times 10^{-1}$	1.08	$7.238\times 10^{-3}$	2.18	$4.787\times 10^{-3}$	2.19
	$4.374\times 10^{-3}$	2.16	$2.039\times 10^{-1}$	1.07	$8.044\times 10^{-2}$	1.05	$1.548\times 10^{-3}$	2.23	$1.016\times 10^{-3}$	2.24
	$1.783\times 10^{-3}$	2.21	$1.321\times 10^{-1}$	1.07	$5.259\times 10^{-2}$	1.05	$6.499\times 10^{-4}$	2.14	$4.235\times 10^{-4}$	2.16
	$7.613\times 10^{-4}$	2.10	$8.287\times 10^{-2}$	1.15	$3.304\times 10^{-2}$	1.15	$2.679\times 10^{-4}$	2.18	$1.715\times 10^{-4}$	2.23
Hexagonal mesh	$3.910\times 10^{-2}$		$7.071\times 10^{-1}$		$2.869\times 10^{-1}$		$1.780\times 10^{-2}$		$1.247\times 10^{-2}$	
	$7.108\times 10^{-3}$	2.46	$3.388\times 10^{-1}$	1.06	$1.368\times 10^{-1}$	1.07	$3.470\times 10^{-3}$	2.36	$2.213\times 10^{-3}$	2.49
	$2.851\times 10^{-3}$	2.25	$2.268\times 10^{-1}$	0.99	$9.127\times 10^{-2}$	1.00	$1.427\times 10^{-3}$	2.19	$9.084\times 10^{-4}$	2.20
	$2.146\times 10^{-3}$	1.84	$1.949\times 10^{-1}$	0.98	$7.840\times 10^{-2}$	0.99	$1.042\times 10^{-3}$	2.04	$6.621\times 10^{-4}$	2.05
	$1.678\times 10^{-3}$	1.86	$1.710\times 10^{-1}$	0.99	$6.870\times 10^{-2}$	1.00	$7.992\times 10^{-4}$	2.00	$5.072\times 10^{-4}$	2.01
Lloyd mesh	$9.223\times 10^{-2}$		$1.084\times 10^{-0}$		$4.440\times 10^{-1}$		$3.715\times 10^{-2}$		$2.365\times 10^{-2}$	
	$1.853\times 10^{-2}$	2.21	$4.744\times 10^{-1}$	1.14	$1.900\times 10^{-1}$	1.17	$7.854\times 10^{-3}$	2.14	$4.781\times 10^{-3}$	2.20
	$8.421\times 10^{-3}$	2.35	$3.303\times 10^{-1}$	1.08	$1.325\times 10^{-1}$	1.07	$3.661\times 10^{-3}$	2.27	$2.227\times 10^{-3}$	2.28
	$4.151\times 10^{-3}$	2.03	$2.328\times 10^{-1}$	1.01	$9.237\times 10^{-2}$	1.04	$1.772\times 10^{-3}$	2.09	$1.139\times 10^{-2}$	1.93
	$2.130\times 10^{-3}$	2.06	$1.729\times 10^{-1}$	0.92	$6.821\times 10^{-2}$	0.93	$9.518\times 10^{-4}$	1.92	$6.057\times 10^{-4}$	1.95

## Data Availability

The data presented in this study are available on request from the corresponding author.

## References

[1] Zhu J., Zeng Q.C. (2008). A mathematical formulation for optimal control of air pollution. Sci. China Ser. D..

[2] Martínez A., Rodríguez C., Vázquez-Méndez M.E. (2000). Theoretical and numerical analysis of an optimal control problem related to wastewater treatment. SIAM J. Control Optim..

[3] Hughes T.J.R., Rooks A. (1982). Streamline upwind/Petrov Galerkin formulations for the convection dominated flows with particular emphasis on the incompressible Navier–Stokes equations. Comput. Methods Appl. Mech. Engrg..

[4] Zhou Z.J., Yan N.N. (2014). A survey of numerical methods for convection-diffusion optimal control problems. J. Numer. Math..

[5] Sun T.J. (2010). Discontinuous Galerkin finite element method with interior penalties for convection diffusion optimal control problem. Int. J. Numer. Anal. Model..

[6] Leykekhman D., Heinkenschloss M. (2012). Local error analysis of discontinuous Galerkin methods for advection-dominated elliptic linear-quadratic optimal control problems. SIAM J. Numer. Anal..

[7] Xu Q.J., Zhou Z.J. (2017). A mixed discontinuous Galerkin approximation of time dependent convection diffusion optimal control problem. J. Math..

[8] Fu H.F., Rui H.X. (2009). A priori error estimates for optimal control problems governed by transient advection-diffusion equations. J. Sci. Comput..

[9] Fu H.F., Rui H.X., Zhou Z.J. (2016). A posteriori error estimates for optimal control problems constrained by convection-diffusion equations. Front. Math. China.

[10] Wang F.Y., Zhang Z.Q., Zhou Z.J. (2021). A spectral Galerkin approximation of optimal control problem governed by fractional advection-diffusion-reaction equations. J. Comput. Appl. Math..

[11] Beirão da Veiga L., Brezzi F., Cangiani A., Manzini G., Marini L.D., Russo A. (2013). Basic principles of virtual element methods. Math. Models Methods Appl. Sci..

[12] Ahmad B., Alsaedi A., Brezzi F., Marini L.D., Russo A. (2013). Equivalent projectors for virtual element methods. Comput. Math. Appl..

[13] Beirão da Veiga L., Brezzi F., Marini L.D., Russo A. (2016). Virtual element method for general second order elliptic problems on polygonal meshes. Math. Models Methods Appl. Sci..

[14] Cangiani A., Manzini G., Sutton O.J. (2017). Conforming and nonconforming virtual element methods for elliptic problems. IMA J. Numer. Anal..

[15] Vacca G., Beirão da Veiga L. (2015). Virtual element methods for parabolic problems on polygonal meshes. Numer. Methods Partial. Differ. Equ..

[16] Antonietti P.F., Beirão da Veiga L., Mora D., Verani M. (2014). A stream virtual element formulation of the Stokes problem on polygonal meshes. SIAM J. Numer. Anal..

[17] Cangiani A., Gyrya V., Manzini G. (2016). The nonconforming virtual element method for the Stokes equations. SIAM J. Numer. Anal..

[18] Benedetto M., Berrone S., Borio A., Pieraccini S., Scialò S. (2016). A hybrid mortar virtual element method for discrete fracture network simulations. J. Comput. Phys..

[19] Berrone S., Borio A. (2017). Orthogonal polynomials in badly shaped polygonal elements for the virtual element method. Finite Elem. Anal. Des..

[20] Manzini G., Cangiani A., Sutton O.J. (2014). The Conforming Virtual Element Method for the Convection-Diffusion-Reaction Equation with Variable Coeffcients.

[21] Benedetto M.F., Berrone S., Borio A., Pieraccini S., Scialò S. (2016). Order preserving SUPG stabilization for the virtual element formulation of advection-diffusion problems. Comput. Methods Appl. Mech. Eng..

[22] Berrone S., Borio A., Manzini G. (2018). SUPG stabilization for the nonconforming virtual element method for advection–diffusion-reaction equations. Comput. Methods Appl. Mech. Eng..

[23] Beirão da Veiga L., Dassi G., Lovadina C., Vacca G. (2020). SUPG-stabilized virtual elements for diffusion-convection problems: A robustness analysis. arXiv.

[24] Talischi C., Paulino G.H., Pereira A., Menezes I.F.M. (2012). PolyMesher: A general-purpose mesh generator for polygonal elements written in Matlab. Struct. Multidiscip. Optim..

[25] Chen L.J., Li M.Z., Xu Q. (2020). Sinc-Galerkin method for solving the time fractional convection-diffusion equation with variable coefficients. Adv. Differ. Equ..

[26] Li L.Y., Jiang Z.W., Yin Z. (2020). Compact finite-difference method for 2D time-fractional convection-diffusion equation of groundwater pollution problems. Comput. Appl. Math..

[27] Zhang C.Y., Liu H.P., Zhou Z.J. (2019). A priori error analysis for time-stepping discontinuous Galerkin finite element approximation of time fractional optimal control problem. J. Sci. Comput..

